# Prevalence and Characteristics of Twitter Posts About Court-Ordered, Tobacco-Related Corrective Statements: Descriptive Content Analysis

**DOI:** 10.2196/12878

**Published:** 2019-10-08

**Authors:** Dannielle E Kelley, Meredith Brown, Alice Murray, Kelly D Blake

**Affiliations:** 1 Cancer Prevention Fellowship Program Division of Cancer Prevention National Cancer Institute Rockville, MD United States; 2 ICF Rockville, MD United States; 3 Health Communication and Informatics Research Branch Division of Cancer Control and Population Sciences National Cancer Institute Rockville, MD United States

**Keywords:** social media, Twitter, tobacco corrective statements, tobacco industry/legislation and jurisprudence

## Abstract

**Background:**

Three major US tobacco companies were recently ordered to publish corrective statements intended to prevent and restrain further fraud about the health effects of smoking. The court-ordered statements began appearing in newspapers and on television (TV) in late 2017.

**Objective:**

The objective of this study was to examine the social media dissemination of the tobacco corrective statements during the first 6 months of the implementation of the statements.

**Methods:**

We conducted a descriptive content analysis of Twitter posts using an iterative search strategy through Crimson Hexagon and randomly selected 19.74% (456/2309) of original posts occurring between November 1, 2017, and March 27, 2018, for coding and analysis. We assessed post volume over time, source or author, valence, linked content, and reference to the industry (eg, big tobacco, tobacco industry, and Philip Morris) and media outlet (TV or newspaper). Retweeted content was coded for source/author and prevalence.

**Results:**

Most posts were published in November 2017, surrounding the initial release of the corrective statements. Content was generally neutral (58.7%, 268/456) or positive (33.3%, 152/456) in valence, included links to additional information about the statements (94.9%, 433/456), referred to the industry (87.7%, 400/456), and did not mention a specific media channel on which the statements were aired or published (15%). The majority of original posts were created by individual users (55.2%, 252/456), whereas the majority of retweeted posts were posted by public health organizations (51%). Differences by source are reported, for example, organization posts are more likely to include a link to additional information compared with individual users (*P*=.03).

**Conclusions:**

Conversations about the court-ordered corrective statements are taking place on Twitter and are generally neutral or positive in nature. Public health organizations may be increasing the prevalence of these conversations through social media engagement.

## Introduction

After an 18-year legal battle, 3 major US tobacco companies (Altria, its subsidiary Philip Morris USA; RJ Reynolds Tobacco; and ITG Brands) were recently ordered by the US District Court for the District of Columbia to publish tobacco corrective statements as 1 of 4 legal remedies originally included in a 2006 judgment to *prevent and restrain* the tobacco companies from continuing to engage in fraud about the harms of cigarettes. These statements were ordered in a federal racketeering lawsuit brought against the tobacco companies in 1999 by the US Department of Justice under the Racketeering Influenced and Corrupt Organizations Act [1-2]. The companies were mandated to publish corrective statements as full-page advertisements in at least 50 newspapers across the country, in 5 issues appearing from late November 2017 to early March 2018, and broadcast statements as 30- and 45-second prime-time spots on major television (TV) networks 5 times a week for 1 year. The statements address 5 areas: (1) adverse health effects of cigarette smoking; (2) adverse health effects of exposure to secondhand smoke; (3) manipulation of cigarette design and composition to enhance nicotine delivery; (4) addictiveness of cigarette smoking and nicotine; and (5) no health benefit from smoking *light*, *low tar*, *ultra light*, *mild*, and *natural* cigarettes.

Since the initial court judgement in 2006, the media landscape has changed substantially. Newspaper readership has declined about 36% [3] and is the least common platform for news consumption [4]. Although TV viewership is still high among older adults, 61% of adults aged 18 to 29 years use streaming services over traditional TV services [5]. In 2006, 16% of US adults reported ever using social media [6], compared with 90% today [7]. Previous studies have found Twitter to be a useful platform for assessing public response to public health topics around tobacco regulation [8], health behaviors [9], and product use [10,11] as 90% of Twitter posts are publicly available from a diverse user base [12]. We examined the prevalence, characteristics, and sources of public Twitter posts about tobacco corrective statements to describe how corrective statements have extended beyond the media outlets in which they were ordered to appear.

## Methods

We used descriptive content analysis to focus on describing the prevalence and characteristics of Twitter posts, without inference to subsequent behavior or message processing [13]. Describing post prevalence and characteristics is an essential step to generating hypotheses and designing future studies to further understand the dissemination and reach of the tobacco corrective statements [14].

### Sample

Crimson Hexagon, a social media analytics tool, was used to collect publicly available Twitter posts related to tobacco corrective statements posted from November 1, 2017, to March 27, 2018. Posts before the release of corrective statements on November 26, 2017, are included as a baseline assessment of changes in post volume. Keywords and search terms were informed by a preliminary review of tobacco corrective statement mentions on social media and in mainstream Web-based news outlets. An iterative refinement process yielded 2309 original posts (as opposed to retweets); 387 (387/ 5167, 7.48%) of the original posts were retweeted a total of 2858 times for a total of 5167 posts during the data interval. Per guidelines set forth by the Department of Health and Human Services Office of Human Research Protections, the data retrieved from Twitter posts set to *public* do not meet the *private and identifiable* standards for personally identifiable information and, therefore, do not meet the definition of human subjects research [15]. See Multimedia Appendix 1 for search process details.

Overall, 19.74% (456/2309) of the original posts were randomly selected for coding and analysis. Furthermore, 90 posts were double coded for interrater reliability by proportion of agreement and Krippendorff alpha. The codebook was refined as necessary. Reliability across constructs ranged from 94% to 100% (Krippendorff alpha .8-1.0), representing moderate-to-perfect agreement.

### Measures

Crimson Hexagon provides descriptive data regarding the volume of posts over time; however, understanding who posted what content is important for contextualizing the communication environment [16]. Posts were coded for source, defined as organization, individual, or undeterminable. To understand post characteristics, the overall tone was coded as positive, positive-unsatisfied (posts in favor of the statements that expressed desire for more corrective action), neutral, and negative. As corrective statements were ordered to appear in newspapers and TV during the data interval of this study, posts were coded for mention of statements in newspapers or on TV. The presence of a link to additional content, and the link’s relevance (relevant/irrelevant) to tobacco corrective statements was coded to understand if users were being referred to additional content [17]. To understand how posts connected the tobacco industry to corrective statements, tobacco industry mentions were coded as no mention, plural (eg, *tobacco industry*), or specific (eg, *Altria*) mention.

To understand post amplification, each of the 387 original posts that were retweeted were further reviewed and categorized by 2 researchers according to the source of the original post: public health organization, news organization, individual, and celebrity.

### Analyses

Frequencies and proportions are reported for the sample of original posts. Chi-square tests were used to understand the differences across sources. Numerical data on retweet prevalence were provided by Crimson Hexagon and analyzed for prevalence by source.

## Results

**Figure 1:**
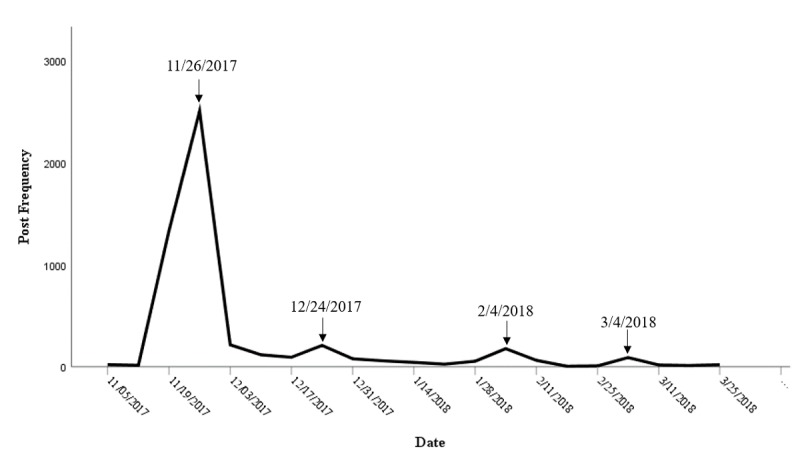
Post prevalence over time.

Of the 5167 posts that appeared between November 1, 2017, and March 27, 2018, 1343 (25.99%) appeared during the week leading up to the release of the corrective statements; 2532 (49.00%) were posted on the day the statements were released. A substantial decrease was observed in the first week of December, and the post volume remained low even after the release of the second and third statements. Modest spikes were observed on the days of the release of the fourth (February 4, 2018) and fifth (March 4, 2018) statements (Figure 1).

Over half (58.7%, 268/456) of the posts were neutral in valence, and 152 of 456 posts (33.3%) were positive or supportive. Most posts (84.8%, 387/456) did not mention a specific media outlet on which the corrective statements were aired or seen. Almost all posts (94.9%, 433/456) contained a link to additional information about the statements, 97.9% (424/433) of which were active and relevant. Furthermore, 87.7% (400/456) of posts referred to the industry generally, whereas less than 1.3% (6/456) mentioned a specific tobacco company. Individual-owned Twitter accounts represented the majority (55.2%, 252/456) of original posts, followed by organization (38.8%, 177/456), and undeterminable accounts (5.9%, 27/456).

Differences across sources were found for media outlet mentioned, presence of link, and industry mentioned. Organizations were more likely to mention that statements appeared in TV and newspapers compared with individual and undeterminable (*χ^2^*_2_=32.6; *P*=.001). Compared with individuals, organizations more often included a link (*χ^2^*_2_=12.7; *P*=.03) and used plural terms to refer to the tobacco industry (*χ^2^*_2_=25.8; *P*=.001), whereas individuals often did not mention the tobacco industry compared with organizations and undeterminable (*χ^2^*_2_=19.7; *P*=.001). The table in Multimedia Appendix 1 describes the post characteristics and differences by source.

Most retweeted posts were from public health organizations (169 posts retweeted 2251 times), followed by individuals (131 posts retweeted 1457 times), news organizations (86 posts retweeted 607 times), and a celebrity (1 post retweeted 126 times).

## Discussion

These results provide a snapshot of the social media conversation around tobacco corrective statements. The presence of posts about these statements demonstrates the public health utility of such platforms for adapting to changes in the communication and regulatory landscape. Considering the drastic changes in the use of the media outlets decided by the court over a decade ago, findings from this study are promising in that most posts were informative or supportive and included links to additional information; thus, the reach of corrective statements may be extended to a broader audience through social media. Consistent with other research that has documented the utility and reach of Twitter for other public health topics [18-22], this study adds evidence to a growing body of literature on Twitter as an important tool for adapting to a dynamic tobacco communication and regulatory environment and for understanding a variety of tobacco-related questions around social conversations about tobacco [23], industry messaging and branding [24-25], tobacco use [10], interventions [9], advocacy [26], and public reactions to tobacco regulation [8]. Findings suggest that public health organizations have been proactive in disseminating tobacco corrective statements and engage with the population in real time. Compared with individual users, organizations maximized information provision by specifying where corrective statements were published, explicitly connecting the tobacco industry to corrective statements, and providing relevant links to additional information.

These findings raise important questions warranting further exploration. First, this study identified the presence of posts about corrective statements and organizations’ potential to expand the reach of these statements beyond the court-ordered media platforms. However, exactly who is exposed to these posts remains unknown. Social network analysis could explore this question and reveal key characteristics such as tobacco-related attitudes and audience affinities to enhance messaging efforts. Second, it is unclear how exposure to corrective statements on social media compares with the court-ordered platforms. Future research could use market research and nationally representative surveys to explore exposure rates across platforms. It is unclear if exposure is associated with tobacco-related attitudes and behaviors and if mode of exposure (ie, social media, TV, and newspaper) modifies such outcomes. Longitudinal studies of exposure and subsequent outcomes are needed.

This study is not without limitations. Data for this study were limited to public Twitter posts, precluding analysis of potential conversations on private accounts. However, over 90% of Twitter accounts are public and accessible through Crimson Hexagon [12] and users tend to be younger and have greater minority representation [7], representing the populations most vulnerable to tobacco use. By analyzing a random subset of posts, the results may not reflect the collective body of corrective statement posts. Smoking behavior was not determinable for most posts, hindering the extrapolation findings to tobacco use behaviors. An inherent limitation to any keyword search strategy is that it is unlikely that all related posts were retrieved. Finally, despite high interrater reliability, our valence code required interpretation, possibly introducing subjective bias.

This snapshot of Twitter conversations about tobacco court-ordered corrective statements indicates that these statements are represented in a larger public information environment that extends beyond traditional media. Continued surveillance of social media responses to corrective statements is warranted to inform public health efforts.

## Appendix

Multimedia Appendix 1 Search process and terms, Preferred Reporting Items for Systematic Reviews and Meta-Analyses (PRISMA) diagram, and table for characteristics of Twitter posts about the corrective statements by post source.

## Abbreviations

NIH: National Institutes of Health
TV: television
