# Supplementary material for: Prevalence and Characteristics of Twitter Posts About Court-Ordered, Tobacco-Related Corrective Statements: Descriptive Content Analysis
Source: JMIR Public Health Surveill. 2019 Oct 8;5(4):e12878. doi: 10.2196/12878 (PMC6806118; doi:10.2196/12878)
Supplement: Multimedia Appendix 1 [file publichealth_v5i4e12878_app1.pdf]

<u>Keyword Search Strategy:</u> Several steps were taken to arrive at the keyword search strategy for this study. First, a preliminary review of mainstream news articles and blog posts regarding the tobacco corrective statements was conducted to obtain the most common phrases used to refer to the corrective statements. For example, CNN ran a story titled: "Big Tobacco's court-ordered ads make their debut" on 11/27/2017, from which we used the terms "court-ordered" and "ad*" as reflected in our keywords in Figure 1 below. This example, and others, were used in addition to "corrective statements" as popular media sources are likely to influence the natural language around the discussion of tobacco corrective statements.

Crimson Hexagon search works similarly to a search with Boolean terms, quotation marks, and truncation. Our first search consisted of inclusion terms only and produced over 30,000 results because the search terms were broad, and many posts were not related to the corrective statements. For example, posts containing both "forced" and "tobacco" were pulled in but were not relevant to the corrective statements. An example of this would be: "Honda should be forced to put a big red/white/black tobacco style warning billboard on every 2017+ CBR."

To focus the search, Crimson Hexagon has a learning algorithm called *Brightview*. To use this algorithm two researchers combed through sections of the returned posts to flag irrelevant posts in Crimson Hexagon and identify common threads between irrelevant posts. For example, many posts were referencing the news of Dr. Brenda Fitzgerald's resignation from her position as CDC director after she was found to be investing in the Tobacco Industry. Therefore, an exclusionary term "CDC Director" was created. After exclusionary terms were identified and irrelevant posts flagged, the search was run again. This process was repeated until most of the posts returned were relevant to the corrective statements and we arrived at the search terms presented in Figure 1 of this supplementary appendix.

Focusing on the sample of Twitter posts, two researchers reviewed the 5,808 posts and manually excluded 641 posts that were not related to the corrective statements and not identified by the algorithm. Of the 5,167 relevant posts, 2,309 were original posts and 2,858 were retweets of 387 original posts. Twenty-percent (n=456) of the original posts were randomly selected for coding, and 90 of these posts were double coded for inter-rater reliability.

Our initial search returned over 30,000 posts across six social media categories. We chose to focus on Twitter for this analysis as this platform represents multiple demographics in terms of age (40% of adults 18-29, 27% of adults 30-49, and 19% of adults age 50-64 use Twitter), race (24% Caucasian, 26% of African Americans and 20% of Hispanics), education (18% high school or less, 25% some college, and 32% college graduates), and location (29% urban, 23% suburban, and 17% rural)[1]. Compared to Instagram and Facebook, most Twitter posts are publicly accessible thereby provide a more complete snapshot of conversations as evidenced by the low number of accessible posts on Instagram and Facebook shown in Figure 2. Finally, forums, blogs, and Reddit cater to subsets of the population who often seek out specific content on these sites, where the focus is the online community or subcommunity rather than the individual user as in sites such as Facebook and Twitter.

1. Pew Research Center (2018). Social Media Fact Sheet. http://www.pewinternet.org/fact-sheet/social-media/. Archived at: www.webcitation.org/746HD3ESn.

Figure 1. Keyword Search Strategy

(Inclusion terms)	("court-ordered" OR "court-mandated" OR "mandated" OR forced OR "compelled" OR "Court-ordered ad*" OR "court-mandated ad*" OR "corrective ad*" OR "corrective statement ad*" OR "corrective statement" OR advertisement OR "court ordered" OR "court mandated")<br>AND<br>("Big Tobacco" OR "Big Tobacco lied" OR "Big Tobacco lying" OR "cigarette compan* lie*" OR "cigarette compan* lying" OR "anti-smoking" OR "anti-smoking ad*" OR Tobacco OR Altria OR Lorillard OR "Philip Morris*" OR "R.J. Reynolds" OR "RJ Reynolds" OR "Reynolds American" OR "Tobacco mea culpa*" OR "Tobacco companies mea culpa*" OR "Big tobacco's mea culpa*" OR "anti-tobacco ad" OR "anti-tobacco ads" OR "anti-tobacco advertisement" OR "anti-tobacco advertisements" OR "anti-tobacco" OR "anti tobacco" OR "Tobacco company" OR "Tobacco companies" OR "anti-smoking" OR "anti smoking")
(Exclusion terms)	AND<br>-(pot OR weed OR marijuana OR cannabis OR "Bureau of Alcohol, Firearms, Tobacco and Explosives" OR "Bureau of Alcohol, Tobacco, Firearms and Explosives" OR UK OR Trump OR "Bureau of Alcohol, Tobacco and Firearms" OR "Bureau of Alcohol, Tobacco, and Firearms")<br>AND<br>-((((chew AND tobacco)~5) OR (chewing AND tobacco)~5) OR "CDC Director" OR "CDC Head" OR fraternity OR pipe OR Japan OR "oh dem poor smokers" OR profit)

Figure 2. PRISMA Diagram Denoting Crimson Hexagon Searches, Inclusions, and Exclusions: Social Media Conversations about Corrective Statements November 1, 2017 – March 27, 2018

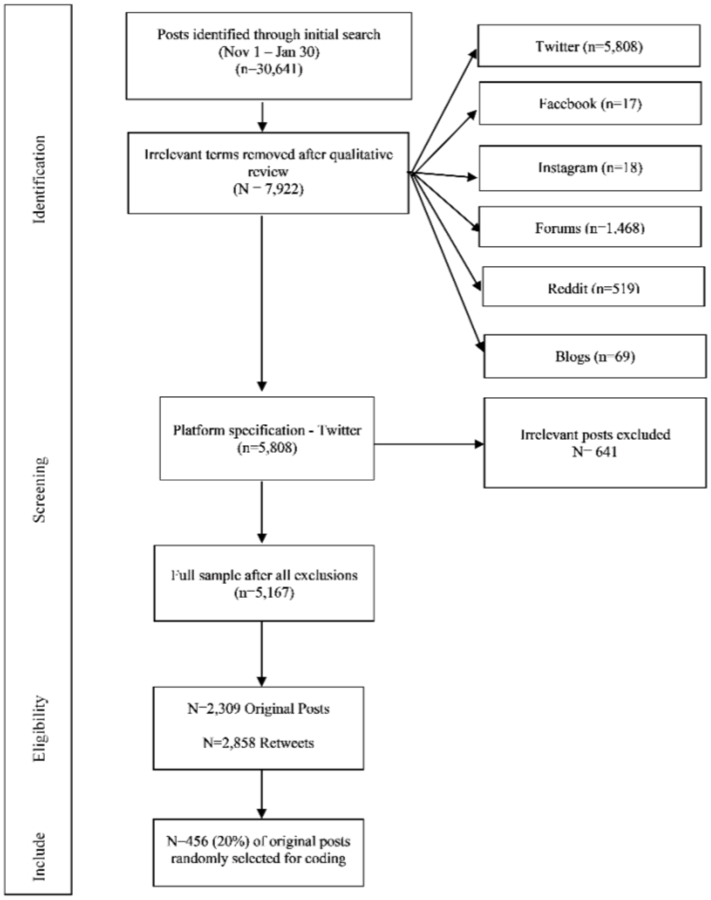

Table 1. Characteristics of Twitter Posts About the Corrective Statements by Post Source

	Organization (n=177)	Individual (n=252)	Unclear (n=27)		Total (N=456)
		N (%)		Chi-Square	N (%)
Valence					
Positive	65 (37)	77 (31)	10 (37)	$\chi^2(2, 456)= 2.10$, *P*=0.35	152 (33)
Positive-unsatisfied	6 (3)	7 (3)	0 (0)	$\chi^2(2, 456)= 0.98$, *P*=0.61	13 (3)
Neutral	100 (57)	152 (60)	16 (59)	$\chi^2(2, 456)= 0.71$, *P*=0.70	268 (59)
Negative	6 (3)	16 (6)	1 (4)	$\chi^2(2, 456)= 1.90$, *P*=0.38	23 (5)
Media outlet					
No mention	135 (76)	225 (89)	27 (100)	$\chi^2(2, 456)=18.90$, *P*=0.001	387 (85)
TV	12 (7)	18 (7)	0 (0)	$\chi^2(2, 456)= 2.00$, *P*=0.36	30 (7)
Newspaper	2 (1)	3 (1)	0 (0)	$\chi^2(2, 456)= 0.32$, *P*=0.85	5 (1)
TV & Newspaper	28 (16)	5 (2)	0 (0)	$\chi^2(2, 456)=32.60$, *P*=0.001	33 (7)
Link					
Yes	175 (99)	231 (92)	27 (100)	$\chi^2(2, 456)=12.70$, *P*=0.03	433 (95)
Industry Mention					
No mention	6 (3)	42 (17)	2 (7)	$\chi^2(2, 456)=19.70$, *P*=0.001	50 (11)
General/non-specific	171 (97)	204 (81)	25 (93)	$\chi^2(2, 456)=25.80$, *P*=0.001	400 (88)
Specific mention	0 (0)	6 (3)	0 (0)	$\chi^2(2, 456)=5.70$, *P*=0.06	6 (1)
